# Adjuvant treatment may benefit patients with high-risk upper rectal cancer: A nomogram and recursive partitioning analysis of 547 patients

**DOI:** 10.18632/oncotarget.10718

**Published:** 2016-07-19

**Authors:** Xin Wang, Jing Jin, Yong Yang, Wen-Yang Liu, Hua Ren, Yan-Ru Feng, Qin Xiao, Ning Li, Lei Deng, Hui Fang, Hao Jing, Ning-Ning Lu, Yu Tang, Jian-Yang Wang, Shu-Lian Wang, Wei-Hu Wang, Yong-Wen Song, Yue-Ping Liu, Ye-Xiong Li

**Affiliations:** ^1^ Department of Radiation Oncology, National Cancer Center/Cancer Hospital, Chinese Academy of Medical Sciences and Peking Union Medical College, Beijing, P. R. China; ^2^ Department of Radiation Oncology, Hunan Cancer Hospital, The Affiliated Cancer Hospital of Xiangya School of Medicine, Hunan, P. R. China

**Keywords:** upper rectal cancer, nomogram, recursive partitioning analysis, adjuvant chemoradiotherapy, adjuvant chemotherapy

## Abstract

**Purpose:**

The role of adjuvant chemoradiotherapy (ACRT) or adjuvant chemotherapy (ACT) in treating patients with locally advanced upper rectal cancer (URC) after total mesorectal excision (TME) surgery remains unclear. We developed a clinical nomogram and a recursive partitioning analysis (RPA)-based risk stratification system for predicting 5-year cancer-specific survival (CSS) to determine whether these individuals require ACRT or ACT.

**Materials and Methods:**

This retrospective analysis included 547 patients with primary URC. A nomogram was developed based on the Cox regression model. The performance of the model was assessed by concordance index (C-index) and calibration curve in internal validation with bootstrapping. RPA stratified patients into risk groups based on their tumor characteristics.

**Results:**

Five independent prognostic factors (age, preoperative increased carcinoembryonic antigen and carcinoma antigen 19-9, positive lymph node [PLN] number, tumor deposit [TD], pathological T classification) were identified and entered into the predictive nomogram. The bootstrap-corrected C-index was 0.757. RPA stratification of the three prognostic groups showed obviously different prognosis. Only the high-risk group (patients with PLN ≤ 6 and TD, or PLN > 6) benefited from ACRT plus ACT when compared with surgery followed by ACRT or ACT, and surgery alone (5-year CSS: 70.8% *vs*. 57.8% *vs*. 15.6%, *P* < 0.001).

**Conclusions:**

Our nomogram predicts 5-year CSS after TME surgery for locally advanced rectal cancer and RPA-based stratification indicates that ACRT plus ACT post-surgery may be an important treatment plan with potentially significant survival advantages in high-risk URC. This may help to select candidates of adjuvant treatment in prospective studies.

## INTRODUCTION

Colorectal cancer (CRC) is one of the most common tumor types worldwide [1]. Total mesorectal excision (TME) and perioperative chemoradiotherapy substantially improved locoregional control for rectal cancer (RC). In China, postoperative adjuvant chemoradiotherapy (ACRT) or adjuvant chemotherapy (ACT) is considered the treatment of choice for stage II or III RC due to the traditional Chinese idea that surgery should be the first therapeutic option.

There are no prospective randomized data for survival outcome based on distance from the anal verge; only subset analysis from randomized trials found a significant correlation between locoregional recurrence and tumor location within the rectum, reporting locoregional recurrence rates of <10% for upper rectal cancer (URC) after TME surgery [2-4], suggesting patients with URC may not significantly benefit from ACRT.

Attempting to determine what patients would benefit from ACRT/ACT, we retrospectively analyzed the data collected from our hospital to construct a clinical nomogram and a recursive partitioning analysis (RPA)-based risk stratification system for predicting 5-year cancer-specific survival (CSS).

## RESULTS

### Baseline patient characteristics and survival

Table 1 summarizes the clinical and pathological characteristics of the 547 patients. The median age was 59 years (range 23-84 years) and the male to female ratio was 1.7:1. The median number of dissected lymph nodes was 14 (range 2-60); the median number of positive lymph nodes was 3 (range 1∼19). The patients were pathologically staged as stage IIa (46.4%), IIb (3.7%), IIIa (2.7%), IIIb (29.3%), and IIIc (17.9%). Median time to ACRT post-surgery was 43 days (range 15-291).

**Table 1:** Clinical characteristics and univariate analysis predicting 5-year CSS rate in patients with URC after TME surgery

Variable	All Cohort		Univariate Analysis			
	*N*	(%)	CSS (%)	HR	95%CI	*P*
Age (years)-Mean, Median, range	58.8, 59.0, 23-84			1.032	1.014-1.051	<0.001
≤40	36	6.6	85.7			
41-50	90	16.5	88.3			
51-60	163	29.8	86.3			
61-70	169	30.9	79.1			
>70	89	16.3	74.1			
CEA (ng/ml)-Mean, Median, range	11.3, 4.2, 0.3-285.4			1.009	0.999-1.019	0.067
≤5	320	58.5	87.9			
>5	227	41.5	75.1			
CA19-9 (U/ml)-Mean, Median, range	35.9, 15.1, 0.3-198.7			1.007	1.002-1.010	<0.001
≤37	419	76.6	85.7			
>37	128	23.4	71.4			
CEA/CA19-9						
CEA^−^/CA19-9^−^	275	50.3	88.3	1(ref.)		
CEA^+^/CA19-9^−^ or CEA-/CA19-9^+^	189	34.6	81.9	1.924	1.211-3.055	0.006
CEA^+^/CA19-9^+^	83	15.2	64.0	3.756	2.271-6.214	<0.001
Distance from the anal verge (cm)^−^ Mean, Median, range	11.6, 11.0, 10.0-16.0			1.030	0.938-1.130	0.537
10-12	392	71.7	81.9			
≥12	155	28.3	84.2			
Tumor size (cm)-Mean, Median, range	4.9, 5.0, 0.5-13.0			1.032	0.926-1.151	0.565
≤5	390	71.3	84.3			
>5	157	28.7	78.2			
Histological differentiation					-	-
High	44	8.0	85.7	1(ref.)		
Moderate	450	82.3	83.1	1.254	0.581-2.709	0.564
Low	53	9.7	74.4	1.380	0.535-3.560	0.506
No. of resected LNs-Mean, Median, range	15.4, 14.0, 2.0-60.0			0.987	0.963-1.010	0.271
≤12	319	58.3	80.7			
>12	228	41.7	83.8			
No. of PLNs-Mean, Median, range	3.9, 3, 1-19.0			2.246	1.471-3.430	<0.001
0	274	50.1	90.4			
1-3	175	32.0	79.2			
4-6	52	9.5	77.8			
≥7	46	8.4	55.0			
Tumor deposit					-	-
Without	441	80.6	86.2	1(ref.)		
With	106	19.4	67.2	2.059	1.343-3.158	<0.001
Pathologic T category						
pT1-3	278	50.8	87.0	1(ref.)	-	-
pT4a	238	43.5	79.4	1.098	0.730-1.652	0.654
pT4b	31	5.7	69.2	2.503	1.361-4.603	<0.001
ACRT						
Without	168	30.7	83.2	1(ref.)		
With	379	69.3	82.3	0.950	0.620-1.453	0.811
ACT						
Without	220	40.2	82.3	1(ref.)		
With	327	59.8	82.6	1.031	0.691-1.536	0.882
Treatment modality						
S alone	92	16.8	80.9	1(ref.)		
S+ACRT or S+ACT	204	37.3	84.3	0.815	0.457-1.455	0.489
S+ACRT+ACT	251	45.9	81.7	0.919	0.530-1.594	0.764

Abbreviations: ACRT, adjuvant chemoradiotherapy; ACT, adjuvant chemotherapy; CA, carcinoma antigen; CEA, carcinoembryonic antigen; CI, confidence interval; CSS, cancer-specific survival; HR, hazard ratio; LNs, lymph nodes; PLNs, positive lymph nodes; S, surgery.

The median follow-up was 68 months (range 4.6-182.5 months) and event rates at 5 years of follow-up were 13.9% for CSS. Until the end of the follow-up, a total of 106 deaths occurred, including 83 (78.3%) from RC, six (5.7%) from other malignancy, 11 (10.4%) from other adverse outcomes (e.g., heart failure, diabetes mellitus, or bowel obstruction) and six (5.7%) from undetermined causes. The actual 5-year overall survival (OS), disease-free survival, and CSS rates were 79.7%, 76.1%, and 83.3%, respectively. Locoregional recurrence and distant metastasis developed in 6.3% and 20.2% of patients, respectively, at five years.

### Adjuvant chemoradiotherapy

External beam radiotherapy (RT), if received, was delivered to the primary tumor bed, anastomosis, and the regional lymphatics by conventional 3-field isocenter radiation (one posterior, two lateral), 3-dimensional conformal radiotherapy, or 5−7-field intensity-modulated radiotherapy with a 6-MV photon beam. The total irradiation dose was 50 Gy in daily 2.0-Gy fractions (five days a week over five weeks). Of the 547 patients, 379 (69.3%) received ACRT.

Among the patients who received ACRT, there was concurrent chemotherapy heterogeneity, which included Capecitabine (*n* = 227, 60.0%), Carmofur (*n* = 61, 16.1%), Doxifluridine (*n* = 46, 12.1%), Tegafur (*n* = 8, 2.1%), and undefined (*n* = 37, 9.8%). Additionally, secondary oxaliplatin was administered in 48% of cases who received oral chemotherapy.

### Adjuvant chemotherapy

Of the 547 patients, 327 (59.8%) received various ACT regimens with a median six cycles (range 1-15) according to oncologist preference, and included FOLFOX (oxaliplatin, 5-fluorouracil [5-FU]/leucovorin; *n* = 115, 35.2%), CapeOX (oxaliplatin, capecitabine; *n* = 69, 21.1%), bolus or infusion 5-FU/leucovorin (*n* = 50, 15.3%), capecitabine (*n* = 29, 8.9%), and undefined (*n* = 64, 19.6%).

### Prognostic nomogram for CSS and internal validation

**Figure 1:**
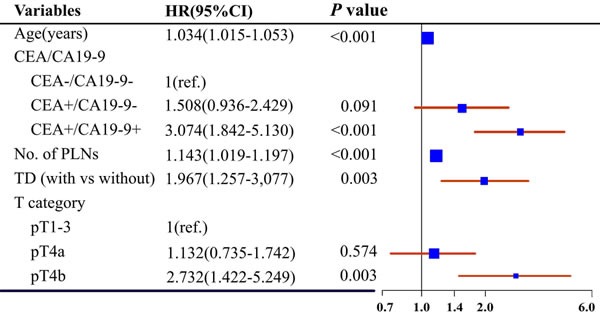
Forest plot of multivariate analysis predicting 5-year CSS The size of the blue box represents the proportion of patients in each subgroup. CA, carcinoma antigen; CEA, carcinoembryonic antigen; CI, confidence interval; CSS, cancer-specific survival; HR, hazard ratio; pT, pathological T classification; PLNs, positive lymph nodes; TD, tumor deposit; -, normal level; +, increased level.

Univariate analysis demonstrated that age, carcinoembryonic antigen [CEA] and carcinoma antigen 19-9 [CA^19^-^9^] category, positive lymph node [PLN], tumor deposit [TD], and pathological T classification were statistically significant predictors of 5-year CSS (Table 1). Interestingly, half of the patients in our study had increased preoperative CEA or CA19-9 levels. Multivariate analysis demonstrated that the five variables were all independent predictors for CSS (Figure 1). The addition of ACRT or ACT or both did not improve CSS for the entire cohort.

**Figure 2:**
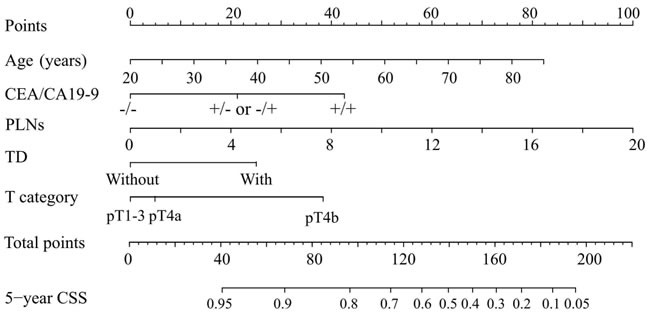
Nomogram predicting 5-year CSS in patients with URC after radical surgery To obtain the nomogram-predicted probability of CSS rate, the patient value at each axis were located. A vertical line was drawn to the “Point” axis to determine how many points could be attributed for each variable value. The points for all variables were summed. Total point values were calculated and then applied to the desired probability scale on the bottom of the Figure. CA, carcinoma antigen; CEA, carcinoembryonic antigen; pT, pathological T classification; PLNs, number of positive lymph nodes; TD, tumor deposit; -, normal level; +, increased level.

**Figure 3:**
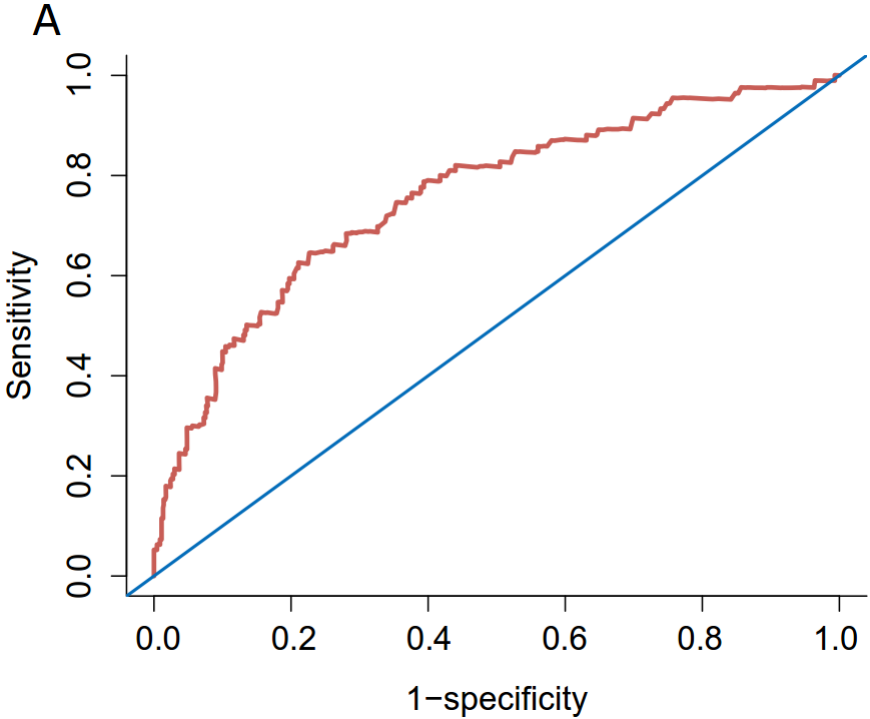
Internal validation of the nomogram predicting 5-year CSS in patients with URC after radical surgery **A.** ROC; C-index = 0.757. **B.** Calibration plot; R^2^ = 0.996. Dotted line (45° line), the ideal line; solid lines, nomogram-predicted probabilities with 95% confidence intervals.

We developed a nomogram based on the five independent predictors of 5-year CSS (Figure 2); the estimated concordance index (C-index) was 0.757 (Figure 3A). The calibration plot for the probability of 5-year CSS post-surgery showed an optimal correlation between the observed and predicted probability. A correlation coefficient, R^2^ = 0.996 was obtained (Figure 3B). The results of the discrimination procedure were established according to criteria 1b of the Transparent Reporting of a Multivariable Prediction Model for Individual Prognosis or Diagnosis (TRIPOD) statement [5] .

### Recursive partitioning analysis

**Figure 4:**
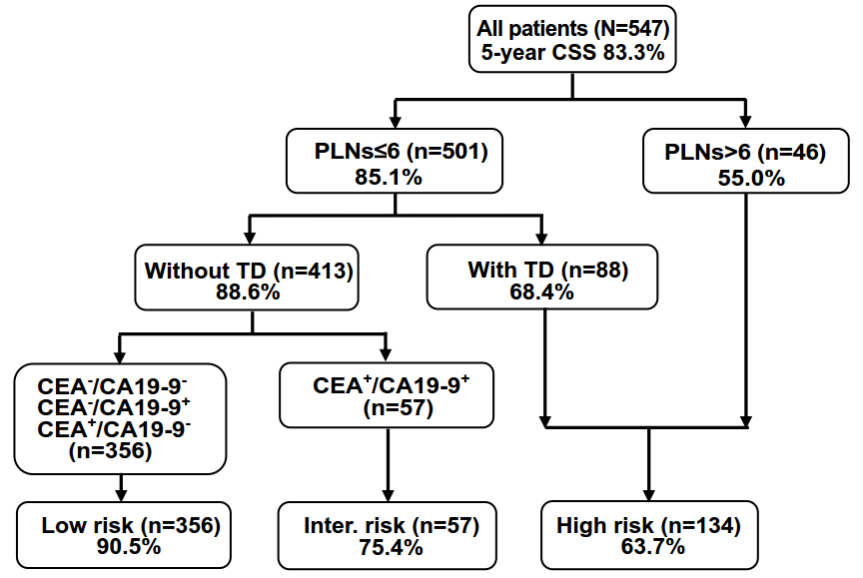
RPA stratification of patients into three risk groups for predicting the 5-year CSS Percentages indicate the 5-year CSS rate. CA, carcinoma antigen; CEA, carcinoembryonic antigen; PLNs, number of positive lymph nodes; TD, tumor deposit; -, normal level; +, increased level.

RPA identified three predictors (PLNs, TD, CEA/CA19-9 category) for stratifying patients according to end point (5-year CSS) and indicated the cutoffs that maximized the separation in risk-specific survival (Figure 4). Patients with >6 PLNs had the poorest 5-year CSS rate (55.0%). Moreover, the 5-year CSS rate in patients with ≤6 PLNs and with TD was unfavorable (68.4%). Accordingly, and considering the small number of cases in these two subsets, we combined them into the high-risk group. Lastly, the patients were conclusively divided into the following risk groups: low (PLNs ≤ 6; without TD; CEA^−^/CA19-9^−^, CEA^+^/CA19-9^−^, or CEA^−^/CA19-9^+^), intermediate (PLNs ≤ 6, without TD, CEA^+^/CA19-9^+^), and high (PLNs ≤ 6 with TD, or PLNs > 6).

**Figure 5:**
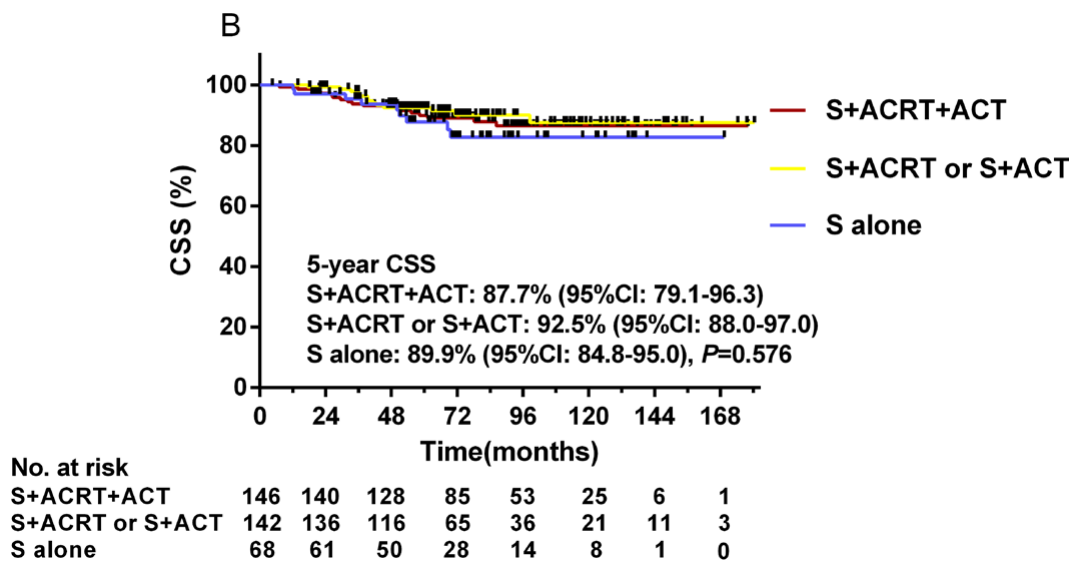
Kaplan-Meier curves depicting CSS in the three RPA-stratified risk groups (A) and in patients treated with different adjuvant treatments in the low-risk group (B), intermediate-risk group (C), and high-risk group (D) **A.** The actual 5-year CSS rate of the low-, intermediate-, and high-risk groups was 90.5%, 75.4%, and 63.7% (*P* < 0.001), respectively. **B.** In the low-risk group, the actual 5-year CSS rates of ACRT plus ACT post-surgery compared to surgery followed by ACRT or ACT, and surgery alone were 87.7%, 92.5% and 89.9% (*P* = 0.576). **C.** In the intermediate-risk group, the actual 5-year CSS rates were 67.8%, 81.2% and 80.0%, respectively (*P* = 0.159). **D.** In the high-risk group, the actual 5-year CSS rates were 70.8%, 57.8% and 15.6%, respectively (*P* < 0.001).

The actual 5-year CSS rate of the low-, intermediate-, and high-risk groups was 90.5%, 75.4%, and 63.7%, respectively. An overall significant difference between groups was evident (*P* < 0.001, Figure 5A).

Stratification according to adjuvant treatment revealed that, in the high-risk group, ACRT plus ACT post-surgery was followed by significantly higher 5-year CSS rates compared to surgery followed by ACRT or ACT, or surgery alone (70.8% *vs*. 57.8% *vs*. 15.6%, *P* < 0.001, Figure 5D). Nevertheless, ACRT and ACT following surgery achieved no significant difference in CSS outcome in the low-risk group (Figure 5B) and intermediate-risk group (Figure 5C).

## DISCUSSION

We developed a clinical nomogram relative to this particular subset of patients with locally advanced URC with complete resection at a single institute. Furthermore, we use RPA to identify sub-stratification, finding that ACRT plus ACT post-surgery was associated with significantly higher 5-year CSS in the high-risk group when compared with surgery alone or surgery followed by ACRT or ACT.

For stage II or III RC, neoadjuvant chemoradiotherapy (NACRT) or RT followed by surgery is associated with considerably decreased local recurrence rate, i.e., <15% [4,6-8]. Despite the disparities in biological behavior between URC and lower RC, the fact remains that the local recurrence rate is 10% and that the 5-year OS of patients with URC is ∼80% [4,6-8]. Therefore, additional attempts at improvement using ACRT or ACT are questionable. In the US, ACRT and ACT are considered the treatment of choice for all patients with stage II or III RC following surgical resection, except those with proximal T3N0 and favorable prognostic features [9]. In Europe, patients with URC with only large tumors with extension to the adjacent structures (T4b) or with multiple metastases in the regional lymph nodes (N2) require NACRT/RT [10]. ACT can also be administered even if the level of scientific evidence for sufficient benefit is much lower than that for stage III and high-risk stage II colon cancer.

In China, surgeon and oncologist decisions might rely on pathological tumor-nodes-metastasis (pTNM) stage and on European and US guidelines in the adjuvant setting. However, there is no consensus on adjuvant treatment of surgically resectable URC to date, and no predictive models have been constructed.

Our study revealed that patients with stage II or III URC had good outcomes after TME surgery, where the 5-year actual OS rate was ∼80% and the locoregional recurrence rate was only 6.3% at five years, which is consistent with the abovementioned study results.

The clinical nomogram incorporates each of the five independent predictors to calculate the 5-year CSS probability. Our findings are highly concordant with previous reports on RC risk factors [11-15]. Interestingly, patients in whom both tumor markers were increased had significantly decreased 5-year CSS compared with patients with increased CEA or CA19-9, or no increase. We cautiously included the two tumor markers as a combined category variable and not two separate continuous variables in the final nomogram because the C-index improved by 0.02 following its addition to the current model. In addition, ACRT and ACT were not selected as candidate factors because univariate analysis revealed that they were not CSS prognostic factors. This is consistent with other study results reporting that not all patients with URC will benefit from adjuvant therapy [16,17]. However, it is worth noting that the nomogram might be unable to identify small subgroups that could benefit from adjuvant treatment.

We also attempted to weigh the risks for the patients. We identified five independent CSS prognostic factors and selected three of them *via* RPA to define three distinct risk groups. Kaplan-Meier survival analysis confirmed three distinct risk groups in which the 5-year CSS rate differed the most. Patients in the high-risk group benefited from ACRT plus ACT as compared with surgery alone or surgery followed by ACRT or ACT (*P* < 0.001). Conversely, no adjuvant modality improved survival in the low- and intermediate-risk groups. In the high-risk group, the number of PLNs was the most robust of the adverse prognostic indicators and affected survival outcome, which many studies have confirmed [15,18,19]. We demonstrated significantly lower survival rates in patients with ≤6 PLNs with TD than in those without TD (68.4% *vs*. 88.6%). TD involves irregular, discrete tumor spread with no evidence of residual lymph node and is thought to arise from lymphovascular invasion. According to the American Joint Committee on Cancer Staging Manual (7^th^ edition) [20], TD is classified as N1c in CRC. Similarly, our results strongly suggest that TD is an independent adverse prognostic factor and it might be a candidate tumor feature for adjuvant settings even though it is only found occasionally in cancer specimens [11,12,21]. Therefore, it appears reasonable in the present study that trimodality treatment (surgery followed by ACRT plus ACT) significantly improved CSS in the high-risk group. The 5-year CSS was excellent for patients in the low-risk group even without ACRT or ACT, suggesting that adjuvant treatment is unnecessary.

For patients in the intermediate-risk group, it is noteworthy that our analysis of results according to treatment method revealed no difference in CSS. That is, although increased CEA and CA19-9 pre-surgery indicated poor prognosis, neither ACRT nor ACT affected the survival outcome of patients in this subgroup. It might be because increased CEA and CA19-9 pre-surgery may be early indicators of tumor recurrence after curative surgery regardless of whether the patient receives adjuvant treatment.

Although there was no statistically significant difference in the 5-year CSS among the different treatment groups, there was a similar trend for decreased 5-year CSS in patients treated with ACRT plus ACT in both the low-risk and intermediate-risk groups. On the one hand, this was most probably because unnecessary and long-term adjuvant treatment may weaken patient immune systems, thereby promoting tumor recurrences. On the other hand, with the fact of lacking survival benefit after the addition of ACRT plus ACT in these patients, the late side effects must be taken into account seriously. There were clearly more common in bowel dysfunction in irradiated patients than in patients who underwent surgery alone in RC [22]. We should also bear in mind the potential impact of those adjuvant treatment on the induction of second malignancies, especially in patients with long-term survival [23,24].

The present study made two contributions. First, we used a nomogram to predict the 5-year CSS in patients with URC following TME surgery. As far as we know, this is the first prognostic model with a relatively high C-index and involving the most cases, specifically focusing on patients with URC. The easy-to-use scoring system allows physicians to perform individualized survival prediction post-surgery. Second, we used RPA to define the risk groups related to 5-year CSS. We determined that high-risk patients might benefit from ACRT plus ACT, which has never been reported in the literature. This aids in the selection of patients who need additional therapy or intensive follow-up and in making clinical treatment decisions. Furthermore, in our setting the analysis of type 1b according to the TRIPOD statement eventually ensures the best modeling procedure to take into account the dataset without splitting the cohort or using different modeling approaches [5].

Our study has some limitations. As a retrospective study, the ACRT/ACT chemotherapy regimens were non-uniform. We also could not obtain information on some important molecular factors (e.g., *KRAS* mutation, *BRAF* mutation, microsatellite instability), records of acute or late complications, and quality of life related to adjuvant treatment. Lastly, our findings should be validated in an external dataset.

In conclusion, patients with stage II or III URC have good survival outcome after TME surgery. Overall, neither ACRT nor ACT or the trimodality combinations improved CSS compared with surgery alone. However, high-risk patients may benefit from ACRT plus ACT. Further external validation will be needed in the future and we will conduct a prospective study to verify our findings.

## MATERIALS AND METHODS

### Patient selection

In 2000-2010, 3995 patients diagnosed with RC were documented in the Chinese Academy of Medical Sciences Cancer Hospital database. The eligibility criteria were as follows: (1) lower edge of the tumor was within 10-16 cm of the anal verge as determined by colonoscopy; (2) patient did not receive NACRT/RT or neoadjuvant chemotherapy (NACT); (3) underwent TME surgery; (4) pathologically proven as stage II or III (American Joint Committee on Cancer Staging Manual, 7^th^ edition [20]); (5) histologic subtype was adenocarcinoma. An eventual 547 patients met the inclusion criteria and were included for the development of the predictive nomogram.

For those cancer patients with good prognosis, such as URC patients with low risk in our study, there is a relatively higher portion of patients dying from conditions other than RC. These findings underscore the limitations of using all deaths, rather than CSS as a measure of treatment. Thus, we examined CSS as the surrogate end point. CSS was defined as the time from surgery to the date of death due to tumor recurrence, or until the last follow-up.

Follow-up including clinical examination, biochemical test, tumor markers, abdominopelvic computed tomography (CT) and chest radiograph and/or CT were performed once every 3 months for 2 years, every 6 months for the next 3 years, and every 12 months thereafter.

As this was a retrospective study, reports of the completeness of the mesorectum removal was not available for all patients and the requirement for informed consent was waived, but hospital ethics committee approved the study (Registration No.: 14-122/912), and it was registered on clinicaltrials.gov.

### Tumor markers

CEA and CA19-9 were all measured in the same laboratory preoperatively. In our study, increased CA19-9 and CEA were defined as >37 U/ml and >5 ng/ml, respectively.

### Statistical analysis

The statistical significance of differences in proportions and medians were compared using independent Wilcox tests for continuous variables and χ^2^ tests for categorical variables. Overall, 8% of patients had missing data, and a multivariate imputation by chained equation procedure was used to deal with the missing data [25].

CSS rates for different variable values were generated using the Kaplan-Meier estimates and compared using the log-rank test. Variables that achieved significance at *P* < 0.05 were entered into multivariable analysis *via* the Cox regression model. Then, a nomogram was developed based on the CSS-predicting Cox regression model. Covariates included age (continuous variable), preoperative serum CEA and CA19-9 categories (no elevations: CEA^−^/CA19-9^−^; increased CEA or CA19-9: CEA^+^/CA19-9^−^ or CEA^−^/CA19-9^+^; increases in both: CEA^+^/CA19-9^+^), the number of PLNs (continuous variable), TD, and pathological T classification. The power of the nomogram was assessed using a C-index estimated by analyzing the area under the curve (AUC) of the receiver operating characteristic (ROC) curve *via* bootstraps with 1000 resamples. Subsequently, the nomogram calibration curve was assessed graphically by plotting the actual proportions against the predicted probabilities.

RPA was performed, incorporating significant predictors identified from the univariate analysis (age, CEA/CA19-9 categories, PLNs, TD, pathological T classification), and modeling 5-year CSS as a dichotomous outcome. Kaplan-Meier curves were used to estimate CSS in each RPA-generated risk group. To discover whether ACRT or ACT affected the patients in each risk group, we reanalyzed CSS according to adjuvant treatment status: surgery alone, surgery with ACRT or ACT, and surgery with ACRT plus ACT.

All statistical analyses were performed using SPSS Version 20.0 (IBM SPSS Inc., Armonk, NY, USA) and the rms, survival ROC, rpart, and forest plot packages in R software Version 3.1.2 (http://www.r-project.org/). All P-values were two-sided; *P* < 0.05 was considered significant.

## References

[1] Torre LA, Bray F, Siegel RL, Ferlay J, Lortet-Tieulent J, Jemal A (2015). Global cancer statistics, 2012. CA Cancer J Clin.

[2] Rosenberg R, Maak M, Schuster T, Becker K, Friess H, Gertler R (2010). Does a rectal cancer of the upper third behave more like a colon or a rectal cancer?. Dis Colon Rectum.

[3] Peeters KC, Marijnen CA, Nagtegaal ID, Kranenbarg EK, Putter H, Wiggers T, Rutten H, Pahlman L, Glimelius B, Leer JW, van de Velde CJ, Dutch Colorectal Cancer Group (2007). The TME trial after a median follow-up of 6 years: increased local control but no survival benefit in irradiated patients with resectable rectal carcinoma. Ann Surg.

[4] Kapiteijn E, Marijnen CA, Nagtegaal ID, Putter H, Steup WH, Wiggers T, Rutten HJ, Pahlman L, Glimelius B, van Krieken JH, Leer JW, van de Velde CJ (2001). Preoperative radiotherapy combined with total mesorectal excision for resectable rectal cancer. N Engl J Med.

[5] Collins GS, Reitsma JB, Altman DG, Moons KG (2015). Transparent reporting of a multivariable prediction model for individual prognosis or diagnosis (TRIPOD): the TRIPOD statement. BMJ.

[6] Improved survival with preoperative radiotherapy in resectable rectal cancer (1997). Swedish Rectal Cancer Trial. N Engl J Med.

[7] Sauer R, Becker H, Hohenberger W, Rodel C, Wittekind C, Fietkau R, Martus P, Tschmelitsch J, Hager E, Hess CF, Karstens JH, Liersch T, Schmidberger H (2004). Preoperative versus postoperative chemoradiotherapy for rectal cancer. N Engl J Med.

[8] Bujko K, Nowacki MP, Nasierowska-Guttmejer A, Michalski W, Bebenek M, Kryj M (2006). Long-term results of a randomized trial comparing preoperative short-course radiotherapy with preoperative conventionally fractionated chemoradiation for rectal cancer. Br J Surg.

[9] NCCN Guidelines Panel (2016). National Comprehensive Cancer Network Guidelines in Oncology (NCCN Guidelines). Rectal cancer.

[10] Schmoll HJ, Van Cutsem E, Stein A, Valentini V, Glimelius B, Haustermans K, Nordlinger B, van de Velde CJ, Balmana J, Regula J, Nagtegaal ID, Beets-Tan RG, Arnold D (2012). ESMO Consensus Guidelines for management of patients with colon and rectal cancer. a personalized approach to clinical decision making. Ann Oncol.

[11] Ueno H, Mochizuki H, Hashiguchi Y, Ishiguro M, Miyoshi M, Kajiwara Y, Sato T, Shimazaki H, Hase K (2007). Extramural cancer deposits without nodal structure in colorectal cancer: optimal categorization for prognostic staging. Am J Clin Pathol.

[12] Lo DS, Pollett A, Siu LL, Gallinger S, Burkes RL (2008). Prognostic significance of mesenteric tumor nodules in patients with stage III colorectal cancer. Cancer.

[13] Stiksma J, Grootendorst DC, van der Linden PW (2014). CA 19-9 As a Marker in Addition to CEA to Monitor Colorectal Cancer. Clin Colorectal Cancer.

[14] Gunderson LL, Jessup JM, Sargent DJ, Greene FL, Stewart A (2010). Revised tumor and node categorization for rectal cancer based on surveillance, epidemiology, and end results and rectal pooled analysis outcomes. J Clin Oncol.

[15] Gunderson LL, Sargent DJ, Tepper JE, Wolmark N, O'Connell MJ, Begovic M, Allmer C, Colangelo L, Smalley SR, Haller DG, Martenson JA, Mayer RJ, Rich TA (2004). Impact of T and N stage and treatment on survival and relapse in adjuvant rectal cancer: a pooled analysis. J Clin Oncol.

[16] Rosenberg R, Maak M, Schuster T, Becker K, Friess H, Gertler R (2010). Does a rectal cancer of the upper third behave more like a colon or a rectal cancer. Dis Colon Rectum.

[17] Popek S, Tsikitis VL, Hazard L, Cohen AM (2012). Preoperative radiation therapy for upper rectal cancer T3,T4/Nx: selectivity essential. Clin Colorectal Cancer.

[18] Valentini V, van Stiphout RG, Lammering G, Gambacorta MA, Barba MC, Bebenek M, Bonnetain F, Bosset JF, Bujko K, Cionini L, Gerard JP, Rodel C, Sainato A (2011). Nomograms for predicting local recurrence, distant metastases, and overall survival for patients with locally advanced rectal cancer on the basis of European randomized clinical trials. J Clin Oncol.

[19] Greene FL, Stewart AK, Norton HJ (2002). A new TNM staging strategy for node-positive (stage III) colon cancer: an analysis of 50,042 patients. Ann Surg.

[20] Edge SB, Byrd DR, Compton CC, Fritz AG, Greene FL, Trotti A (2010). AJCC cancer staging manual.

[21] Nagayoshi K, Ueki T, Nishioka Y, Manabe T, Mizuuchi Y, Hirahashi M, Oda Y, Tanaka M (2014). Tumor deposit is a poor prognostic indicator for patients who have stage II and III colorectal cancer with fewer than 4 lymph node metastases but not for those with 4 or more. Dis Colon Rectum.

[22] Peeters KC, van de Velde CJ, Leer JW, Martijn H, Junggeburt JM, Kranenbarg EK, Steup WH, Wiggers T, Rutten HJ, Marijnen CA (2005). Late side effects of short-course preoperative radiotherapy combined with total mesorectal excision for rectal cancer: increased bowel dysfunction in irradiated patients—a Dutch colorectal cancer group study. J Clin Oncol.

[23] Birgisson H, Påhlman L, Gunnarsson U, Glimelius B (2005). Occurrence of second cancers in patients treated with radiotherapy for rectal cancer. J Clin Oncol.

[24] de Gonzalez A, Curtis RE, Kry SF, Gilbert E, Lamart S, Berg CD, Stovall M, Ron E (2011). Proportion of second cancers attributable to radiotherapy treatment in adults: a cohort study in the US SEER cancer registries. Lancet Oncol.

[25] van Buuren S, Groothuis-Oudshoorn K (2011). Mice: Multivariate imputation by chained equations in r. Journal of statistical software.

